# Attitudes and behaviors toward hypnosis and hypnotic susceptibility in physiotherapy patients: a cross-sectional study

**DOI:** 10.3389/fpsyg.2026.1766305

**Published:** 2026-03-05

**Authors:** Ozlem Kuculmez, Emine Dundar Ahi, Ilhan Celil Ozbek, Sevgi Ikbali Afsar

**Affiliations:** 1 Department of Physical Medicine and Rehabilitation, Alanya Hospital, Baskent University, Antalya, Türkiye; 2 Department of Physical Medicine and Rehabilitation, Kocaeli Health and Technology University, Kocaeli, Türkiye; 3 Department of Physical Medicine and Rehabilitation, Derince Training and Research Hospital, University of Health Sciences, Kocaeli, Türkiye; 4 Faculty of Medicine, Department of Physical Medicine and Rehabilitation, Baskent University, Ankara, Türkiye

**Keywords:** attitude, awareness, behavior, complementary therapies, hypnosis, knowledge, physical therapy

## Abstract

**Background and objectives:**

Hypnosis is a focused cognitive state with increased suggestibility and neurophysiological changes, but misconceptions remain despite its clinical value. This study examined physiotherapy patients' beliefs and behaviors about hypnosis, their hypnotic suggestibility levels, and the relationships among these factors.

**Materials and methods:**

This cross-sectional study included voluntary adult participants aged 18–70 years who presented to the physical medicine and rehabilitation outpatient clinics. Individuals with psychiatric disorders, cognitive impairment, poor general health, illiteracy, or incomplete data were excluded. Demographic characteristics were recorded, and participants were asked to complete the attitudes and beliefs toward hypnosis scale (VSABTH-T) and the Short Form of the Suggestibility Scale (SSS). The study evaluated participants' attitudes, beliefs, and levels of suggestibility, as well as the relationships among these variables. A significance level of *p* < 0.05 was accepted for all statistical analyses.

**Results:**

The study analyzed data from 399 participants (65.9% male, 34.1% female) with a mean age of 42.68 ± 16.75 years. 75.4% of participants had limited knowledge, and 3.5% had hypnosis experience. Predominantly expressed concerns regarding hypnotic procedures were 86.2% fear, 61.9% emerging from hypnosis, 51.8% losing control, and 33.3% of the participants defined hypnosis as dangerous. Participants had a mean attitude and behavior score toward hypnosis of 107.21 ± 15.8 and a mean hypnotizability score of 53.29 ± 8.76. Although age, occupation, and income level showed very weak but statistically significant correlations with the total VSABTH-T scores (*p* = 0.001), there was no correlation with total SSS scores (*p* > 0.05). Attitudes toward hypnosis and hypnotic suggestibility are significantly affected by age, education, occupation, income, and particularly the level of knowledge about hypnosis (*p* < 0.05). Total VSABTH-T and SSS scores were not correlated (*p* = 0.069), although very weak associations were observed between subdimensions (*p* < 0.05).

**Conclusion:**

Physiotherapy patients demonstrated limited hypnosis knowledge and experience and misconceptions. Demographic factors showed weak associations with attitudes and suggestibility. Findings highlight that personal beliefs and demographics interact complexly in shaping hypnotic responsiveness, emphasizing the need for targeted education to reduce misconceptions.

## 1 Introduction

Hypnosis may be described as a form of guided interaction through which an individual shifts into a modified level of awareness. In this condition, attention becomes highly focused while the body simultaneously reaches a state of profound calm and relaxation. Throughout the induction process, attention is gently moved away from external stimuli and redirected toward internal experiences. This inward focus makes it easier to engage with meaningful internal thoughts and sensations in a calm and attentive way. Therapeutic guidance is used to reinforce this internal exploration, helping the individual maintain attention on relevant inner experiences. As a result, learning can occur beyond conscious reasoning, involving emotional responses and bodily processes. Additionally, the natural reduction of stress during hypnotic states supports emotional balance and physiological regulation (Geagea et al., 2024, 2023a). With technological advances, magnetic resonance and electroencephalography monitoring have become effective tools for observing and interpreting the changes that hypnosis produces in brain structures. Research has shown that medial, prefrontal, and anterior cingulate cortices are affected during hypnotic interventions (Terhune et al., 2017).

Hypnosis is used in the treatment of hyperemesis gravidarum, nocturnal enuresis, sleep and eating disorders, obesity, depression and anxiety disorders, smoking addiction, warts and allergic skin manifestations, phobias, and many painful conditions. In addition, it is applied as an effective method across a wide range of areas, from reducing pain during medical interventions and alleviating emotional stress to improving irritable bowel syndrome symptoms and providing suggestion-based relief in various medical procedures. Meta-analyses have shown significant results, particularly in pain management and IBS, and suggestion techniques applied without formal trance induction have also been reported to be beneficial in medical settings (Williamson, 2019; Häuser et al., 2016; Shenefelt, 2018; Chiu et al., 2018). Several studies have investigated the use of hypnosis to alleviate dental anxiety and anxiety associated with dental surgery (Wolf et al., 2022; Leonan-Silva et al., 2025). Häuser et al. suggested in their mini-review that gut-directed hypnosis may be a viable intervention for irritable bowel syndrome in selected patients (Häuser, 2024). Pellegrini et al. in their review, stated that hypnosis can be used alongside diet and physical activity for obesity management, while emphasizing the need for further research (Pellegrini et al., 2021). In the meta-analysis conducted by Thompson et al., hypnosis was found to significantly reduce pain levels, indicating that it is a reliable and effective intervention (Thompson et al., 2019). Geagea et al. suggested that hypnosis may have potential benefits in reducing pain in children; however, they emphasized that further studies are required (Geagea et al., 2023b). All these findings indicate that hypnosis is increasingly gaining attention as a complementary therapeutic method, and scientific research in this field continues to advance steadily.

One of the major challenges in the field of hypnosis is the prevalence of prejudices and misconceptions about it among both patients and healthcare professionals. Hypnosis is not a process in which a person loses self-control, becomes unaware, or cannot remember what happened, nor is it a mental state where suggestions are automatically accepted without question. During hypnosis, the individual does not lose awareness or consciousness but becomes less responsive to external stimuli (Taştan et al., 2015). Hypnosis may not be considered a therapy on its own; however, like a syringe that facilitates the injection of medication, it can serve as a tool that helps deliver treatment more effectively. Hypnosis does not make the impossible possible, but it can help patients believe in themselves and experience what they are capable of achieving (Tastan et al., 2018).

In a study investigating patients' and relatives' knowledge, beliefs, and attitudes toward the use of hypnosis for sedation and analgesia in the emergency department, 384 participants were included. The results showed that participants were mostly influenced by incorrect information sources and therefore held strong prejudices and misconceptions about hypnosis, leading to reluctance to prefer it in most clinical scenarios (Güneş and Serhat, 2021). Another study aimed to evaluate healthcare professionals' attitudes and beliefs toward hypnosis using the Valencia Scale, including 148 participants from various medical roles. The findings demonstrated that misconceptions and stereotypical beliefs about hypnosis were still common among clinicians, highlighting significant fear, control concerns, and misunderstandings about memory and magical notions. The authors concluded that increasing accurate knowledge is essential to reduce prejudice and promote wider clinical use of hypnosis in areas such as pain and anxiety management (Hegde and Mutalik, 2022).

One of the most well-established findings from hypnosis research is that individuals show considerable variability in their responsiveness to hypnotic suggestions. Since this variability affects both treatment outcomes in therapeutic applications of hypnosis and responses to suggestions in experimental settings, it is important for clinicians and researchers to use robust instruments to measure hypnotic suggestibility. There are standardized tests designed to determine levels of hypnotic susceptibility (Thompson et al., 2019). There are many clinical studies investigating the effects of hypnosis as a supportive treatment (Hegde and Mutalik, 2022; Acunzo and Terhune, 2021; Jones et al., 2024). However, when the literature was reviewed, we did not find any studies specifically examining the awareness of hypnosis among physical therapy and rehabilitation patients. The aim of this study is to evaluate attitudes and behaviors toward hypnosis and the level of hypnotic susceptibility in physiotherapy patients and to determine the relationship between them.

## 2 Materials and methods

This was a cross-sectional study consisting of individuals presenting to the Physical Medicine and Rehabilitation Outpatient Clinics of Baskent University and Derince Research and Training Hospital who voluntarily provided informed consent. Its ethical approval was obtained from the Ethics Committee of Baskent University Faculty of Medicine (KA25/432, approval no: E-94603339-604.02-528764, approval date: 10.11.2025), and all procedures conducted in this study adhered to the ethical principles outlined in the Declaration of Helsinki.

### 2.1 Sample size and patient selection

Sample size calculations were performed using the G^*^Power 3.1.9.4 software. Assuming a 95% confidence level, α = 0.05, a margin of error (*d*) = 0.05, and an expected proportion of *p* = 0.50, the minimum required sample size for the study was estimated to be 384 participants.

Male and female patients aged 18–70, patients presenting to the Physical Medicine and Rehabilitation outpatient clinics for examination, and individuals who have been informed about the cross-sectional study and have provided consent to participate were included in the study. Participants with any psychiatric disorder, poor general health status, or uncontrolled systemic disease; who are illiterate; whose cognitive level is not sufficient to understand questions related to hypnosis; and who provided incomplete data were excluded from the study.

### 2.2 Evaluation of the participants

All patients who attended the physical medicine and rehabilitation polyclinic were evaluated by a physician, and demographic details such as age, gender, educational status, occupation, income level, and previous experience with hypnosis were recorded. Afterwards, participants were asked to complete the Turkish version of the Attitudes and Beliefs Toward Hypnosis Scale and the Turkish version of the Short Form of the Suggestibility Scale. The attitudes and beliefs of the participants toward hypnosis and the degree of suggestibility, as well as the relationship between these variables, were statistically analyzed.

The Turkish validity and reliability study of the Valencia scale of attitudes and beliefs toward hypnosis (VSABTH-T) was conducted as a thesis in the Department of Family Medicine, and the related article is currently in the publication process; permission was obtained from the author to use the scale in this study (Koçak, 2023). It was first developed by Capafons et al. in 2015 to assess individuals' attitudes and beliefs toward hypnosis, which consisted of 37 items and 8 subscales (Capafons et al., 2015). The Turkish version was consisted 33 items and 7 subscales, including help and memory, fear, control, interest, magical, cooperation, and marginal. Help refers to the belief that hypnosis enhances therapeutic effects and improves medical-psychological interventions; memory reflects the idea that a person under hypnosis tells the truth and can reveal memories they cannot otherwise recall; fear involves the misconception that hypnosis carries risks and dangers, including the belief that individuals may become trapped in hypnosis; control denotes the understanding that a person under hypnosis maintains control over their actions and can resist suggestions if they choose; interest represents the desire to be hypnotized or to be highly hypnotizable; magical refers to the belief that suggestions given during hypnosis can solve or correct problems without requiring any effort from the individual; cooperation emphasizes that becoming hypnotized requires the individual's effort and participation, and marginal beliefs include the misconceptions that hypnosis is unrelated to scientific research and that individuals under hypnosis are psychologically abnormal. Items in the scale are rated using a six-point Likert scale (1 = strongly disagree, 2 = disagree to a great extent, 3 = disagree, 4 = agree, 5 = agree to a great extent, 6 = strongly agree). The scale accounted for a total explained variance of 60.682%. The internal consistency analysis of the 33-item instrument yielded a Cronbach's alpha coefficient of 0.819 (Koçak, 2023).

The Short Form of the Suggestibility Scale (SSS) was first developed by Kotov et al. with the name of the Multidimensional Iowa Suggestibility Scale (MISS). The MISS was a 91-item scale composed of five subdimensions (Kotov et al., 2004). The Turkish validity and reliability study of this scale was conducted in 2023 by Koroglu et al. This scale was consisted 21 items and five subdimensions, including peer conformity, physiological suggestibility, persuadability, consumer suggestibility, and influence of others. Items in the scale are rated using a five-point Likert scale (1 = never, 2 = rarely, 3 = sometimes, 4 = often, 5 = always). Reliability analysis yielded a Cronbach's alpha of 0.87 for the under-18 group and 0.86 for the adult group. Construct validity was supported through exploratory factor analysis (EFA), which revealed five factors accounting for 52.59% of the total variance. Confirmatory factor analysis (CFA) demonstrated satisfactory model fit indices. The total score of the Brief Suggestibility Scale showed significant correlations with the autonomous self (*r* = −0.43) and relational self (*r* = 0.31) subscales. Overall, the scale demonstrated strong internal consistency (α = 0.86) and high test–retest reliability (*r* = 0.85, *p* < 0.01) (Köroglu, 2022).

Following the evaluation, the participants' attitudes and behaviors toward hypnosis, their likelihood of being hypnotized, and the relationships among these variables were statistically examined.

## 3 Statistical analysis

All statistical procedures in this research were carried out with IBM SPSS Statistics 21.0 (IBM Corp., Armonk, NY, USA). Continuous variables were summarized using both mean ± standard deviation and median values with their respective ranges. When the data satisfied the assumptions of normality and homogeneity of variance, comparisons between two groups were conducted using the independent samples Student's *t*-test, while comparisons among more than two groups were performed using one-way analysis of variance (ANOVA). When ANOVA indicated a significant difference among group means, pairwise post hoc comparisons were carried out using the Bonferroni correction method. In cases where the assumptions required for parametric tests were not met, the Mann–Whitney *U*-test was used for comparisons between two groups, and the Kruskal–Wallis *H* test was applied for comparisons involving more than two groups. The strength and direction of the association between two quantitative variables were assessed using Pearson correlation analysis. To evaluate the effects of independent variables on the dependent variable, as well as the direction and predictive capacity of these relationships, multiple linear regression analysis (multivariate linear regression) was employed according to the type of dependent variable. For all statistical analyses, a significance level of *p* < 0.05 was considered.

## 4 Results

A total of 426 individuals initially participated in the study; however, 26 were excluded due to incomplete data, resulting in an analytic sample of 399 participants. The mean age of the participants was 42.68 ± 16.75 years, and most of them were male. It was found that the majority of participants were civil servants, health professionals, housewives, or retirees. Most participants were high school graduates and reported having a low income. More than 75% of the participants reported having no or limited prior knowledge of hypnosis, and only 3.5% indicated that they had previously experienced hypnosis. Additionally, 63.9% of participants (*n* = 255) reported that they did not wish to undergo hypnosis. Among the participants, 86.2% (*n* = 344) reported experiencing fear related to hypnosis, 61.9% (*n* = 247) expressed concern about being unable to emerge from a hypnotic state, 51.8% (*n* = 207) were worried about losing control during hypnosis, and 33.3% (*n* = 133) perceived hypnosis as dangerous. On the other hand, 32.5% (*n* = 130) of the participants regarded hypnosis as useful, and 9.7% (*n* = 39) considered it safe. The mean attitude and behavior score toward hypnosis among the participants and detailed demographic characteristics of the participants were presented in Table 1.

**Table 1:** Demographic features of the participants.

**Parameter**		
Age (years; mean ± SD)	42.68 ± 16.75	
Gender *n* (%)	Male	263 (65.9)
	Female	136 (34.1)
Education *n* (%)	Primary school	10 (2.5)
	Secondary school	47 (11.8)
	High school	171 (42.9)
	University	143 (35.8)
	Master's degree	28 (7.0)
Occupation *n* (%)	Health professional	88 (22.1)
	Officer	85 (21.3)
	Unemployed	81 (20.3)
	Retired	81 (20.3)
	Worker	24 (6.0)
	Manager	21 (5.3)
	Tradesman	18 (4.5)
	Academician	1 (0.3)
Economic income *n* (%)	Low	197 (49.4)
	Intermediate	147 (36.8)
	High	55 (13.8)
Hypnosis knowledge level *n* (%)	None	125 (31.3)
	Low	176 (44.1)
	Intermediate	95 (23.8)
	High	3 (0.8)
Hypnosis experience *n* (%)	Yes	14 (3.5)
	No	385 (96.5)
Attitude and behavior score toward hypnosis (mean ± SD)	107.21 ± 15.8	
Hypnotizability score (mean ± SD)	53.29 ± 8.76	

Age was negatively correlated with VSABTH-T total score (*r* = −0.201, *p* = 0.001) and with the fear (*r* = −0.214, *p* < 0.001), control (*r* = −0.237, *p* = 0.001), and cooperation (*r* = −0.152, *p* = 0.002) subscales. No significant correlation was found between age and total SSS score (*r* = 0.094, *p* = 0.062); however, age was positively correlated with physiological suggestibility (*r* = 0.13, *p* = 0.009).

Gender and education level were not associated with VSABTH-T or SSS total scores (*p* > 0.05). Occupational comparisons showed that officers had higher cooperation, fear, help-memory, and total attitude scores (all *p* = 0.001), while unemployed participants had higher control scores (*p* = 0.001). Regarding SSS subdimensions, unemployed participants demonstrated higher physiological suggestibility (*p* = 0.001), and officers showed higher persuasibility (*p* = 0.024), with no difference in total SSS scores (*p* = 0.231).

By income level, low-income participants had higher physiological suggestibility (*p* = 0.002), and high-income participants had higher cooperation scores (*p* = 0.001). Total SSS scores did not differ by income (*p* = 0.303), whereas total VSABTH-T scores were higher in the middle-income group (*p* = 0.018).

Hypnosis knowledge and prior experience were not associated with VSABTH-T or SSS total scores; the only significant difference was observed in the help-memory subscore (*p* = 0.038) (Tables 2, 3). No significant correlation was found between total VSABTH-T and total SSS scores (*p* = 0.069). At the subscale level, physiological suggestibility showed weak negative correlations with control (*r* = −0.011, *p* = 0.029) and cooperation (*r* = −0.168, *p* = 0.001). Persuasibility showed weak positive correlations with control (*r* = 0.107, *p* = 0.033) and interest (*r* = 0.101, *p* = 0.043). Cooperation was weakly negatively correlated with total SSS score (*r* = −0.099, *p* = 0.049; Table 4).

**Table 2:** Comparisons of hypnosis knowledge level, VSABTH-T and SSS scores.

**Hypnosis knowledge level**	**None (min-max)**	**Low (min-max)**	**Intermediate (min-max)**	***p* value ^μ^**
Peer conformity	7 (6–8)	7 (6–8)	7 (6–8)	0.832
Physiological suggestibility	14 (11–16)	14 (11–16)	14 (12–16)	0.438
Persuadability	14 (13–16)	14 (13–15)	14 (12–15)	0.113
Consumer suggestibility	9 (8–11)	9 (8–10)	9 (7.5–10)	0.164
Influence of others	8 (7–9)	8 (7–9)	8 (7–9)	0.29
Total SSS score	52 (49–57)	52 (48–56)	53 (46–55.5)	0.652
Help and memory	33 (30–37)	34 (31.75–37)	34 (32–37)	0.465
Fear	17 (14–20)	16 (13–20)	15 (14–19)	0.337
Control	19 (17–22)	19 (17–21)	18 (17–21)	0.461
Interest	13 (12–14)	13 (12–13)	13 (12–14)	0.3
Magical	9 (8–12)	9 (7–11)	9 (7–10.5)	0.3
Cooperation	11 (9–12)	11 (9–12)	11 (10–13)	0.106
Marginal	8 (7–9)	8 (7–9)	8 (7–8)	0.583
VSABTH-T total score	109 (99–116)	107 (99–116.25)	107 (99–115)	0.905

^μ^ Kruskal Wallis Test.

^*^p < 0.05 statistically significant.

SSS, the short form of the suggestibility scale; VSABTH-T, Valencia scale of attitudes and beliefs toward hypnosis.

**Table 3:** Comparisons of hypnosis experience, VSABTH-T and SSS scores.

**Hypnosis experience**	**No experience (min-max)**	**Experienced (min-max)**	***P* value ^μ^**
Peer conformity	7 (6–8)	7.5 (7–9)	0.066
Physiological suggestibility	14 (11–16)	14 (12.25–15.75)	0.637
Persuadability	14 (13–16)	15.5 (13.5–17)	0.153
Consumer suggestibility	9 (8–10)	9 (8–10.75)	0.861
Influence of others	8 (7–9)	8 (7–10.25)	0.868
Total SSS score	52 (48–56)	53.5 (50.5–59.75)	0.247
Help and memory	34 (31–37)	35 (34–40)	**0.038** ^*^
Fear	17 (14–20)	15 (13.25–19.75)	0.504
Control	18 (17–22)	21 (18.5–24)	0.148
Interest	13 (12–14)	13 (11–14.75)	0.693
Magical	9 (7–11)	9 (7.25–10.75)	0.656
Cooperation	11 (9–12)	13 (9.25–14.75)	0.108
Marginal	8 (7–9)	8 (7.25–8.75)	0.841
VSABTH-T total score	107 (99–116)	110.5 (104.5–117.75)	0.259

^μ^ Mann Whitney U-Test.

^*^p < 0.05 statistically significant.

SSS, the short form of the suggestibility scale; VSABTH-T, Valencia scale of attitudes and beliefs toward.

**Table 4:** Pearson's correlation coefficients (*r*) and *p*-values between evaluation metrics for the total VSABTH-T, SSS scores and subscores.

**Parameter**	***p* value**	**Peer conformity**	**Physiological suggestibility**	**Persuadability**	**Consumer suggestibility**	**Influence of others**	**Total SSS score**
Help and memory^μ^	*r*	0.045	−0.084	0.025	−0.07	0.003	−0.043
	*p*	0.375	0.094	0.622	0.163	0.958	0.387
Fear^μ^	*r*	−0.049	−0.047	−0.066	−0.083	−0.066	−0.079
	*p*	0.333	0.351	0.19	0.096	0.187	0.117
Control^μ^	*r*	0.017	−0.11	0.107	−0.071	−0.01	−0.043
	*p*	0.73	**0.029** ^*^	**0.033** ^*^	0.154	0.844	0.387
Interest^μ^	*r*	0.044	0.015	0.101	−0.007	0.049	0.047
	*p*	0.376	0.761	**0.043** ^*^	0.883	0.328	0.344
Magical^μ^	*r*	−0.041	−0.093	−0.048	−0.081	−0.034	−0.09
	*p*	0.414	0.064	0.343	0.105	0.5	0.071
Cooperation^μ^	*r*	0.019	−0.168	0.029	−0.063	−0.063	−0.099
	*p*	0.701	**0.001** ^*^	0.562	0.207	0.207	**0.049** ^*^
Marginal^μ^	*r*	0.003	−0.048	0.049	0.015	0.016	−0.006
	*p*	0.951	0.34	0.333	0.763	0.754	0.909
VSABTH-T total score^μ^	*r*	0	−0.135	0.024	−0.096	−0.043	−0.091
	*p*	0.995	0.007	0.024	0.055	0.396	0.069

^μ^ Pearson's correlation test.

^*^p < 0.05 statistically significant.

SSS, the short form of the suggestibility scale; VSABTH-T, Valencia scale of attitudes and beliefs toward hypnosis.

In regression analyses predicting VSABTH-T scores, knowledge about hypnosis positively predicted help-memory (*B* = 5.692, *p* = 0.038), control (*B* = 8.055, *p* = 0.003), and cooperation (*B* = 0.723, *p* = 0.034). Age negatively predicted fear (*B* = −0.060, *p* < 0.001), control (*B* = −0.072, *p* < 0.001), and cooperation (*B* = −0.025, *p* = 0.005). Officers (*B* = 4.375, *p* = 0.021) and healthcare professionals (*B* = 1.621, *p* = 0.006) demonstrated more positive attitudes, whereas workers showed lower scores in some dimensions (*B* = −1.526, *p* = 0.014). Education level was associated with fear (*B* = −0.957, *p* = 0.034) and total VSABTH-T score (*B* = 3.456, *p* = 0.033; Table 5).

**Table 5:** Multiple regression models predicting VSABTH-T scores.

**Dependent variable**	**Predictors**	**B**	**SE**	***t***	***p*-value**	**Vif**	**Tolarence**
Help and memory	(Intercept)	33,227	0,312	106,515	< 0.001		
	Occupation (officer)	1,961	0,596	3,289	0,001	1,08	0,92
	Occupation (health professional)	1,621	0,591	2,743	0,006	1,09	0,92
	Hypnosis knowledge (high)	5,692	2,728	2,086	0,038	1,01	0,99
	Model fit: adjusted *R*^2^ = 0.0395, *F*_(3, 395)_ = 6.45, *p* value = < 0.001						
Fear	(Intercept)	20,061	0,687	29,184	< 0.001		
	Age	−0,060	0,013	−4,535	< 0.001	1,03	0,97
	Income level (high)	1,386	0,646	2,146	0,032	1,04	0,96
	Education (high school)	−0,957	0,450	−2,127	0,034	1,04	0,96
	Model fit: adjusted *R*^2^ = 0.08, *F*_(4, 394)_ = 9.66, *p* value = < 0.001						
Control	(Intercept)	22,242	0,646	34,408	< 0.001		
	Age	−0,072	0,014	−5,179	< 0.001	1,01	0,99
	Hypnosis knowledge (high)	8,055	2,688	2,996	0,003	1,00	1,00
	Occupation (Worker)	−2,345	0,981	−2,391	0,017	1,01	0,99
	Model fit: adjusted *R*^2^ = 0.0837, *F*_(3, 395)_ = 13.12, *p* value = < 0.001						
Magical	(Intercept)	9,250	0,142	64,924	< 0.001		
	Income level (high)	−0,777	0,384	−2,025	0,043		
	Model fit: adjusted *R*^2^ = 0.0077, *F*_(1, 397)_ = 4.1, *p* value = 0.0435						
Cooperation	(Intercept)	10,989	0,450	24,409	< 0.001		
	Income level (intermediate)	1,144	0,319	3,586	< 0.001	1,19	0,84
	Income level (high)	0,936	0,436	2,149	0,032	1,14	0,88
	Occupation (Memur)	0,944	0,357	2,648	0,008	1,08	0,93
	Occupation (worker)	−1,526	0,617	−2,472	0,014	1,09	0,92
	Age	−0,025	0,009	−2,810	0,005	1,09	0,92
	Hypnosis knowledge (intermediate)	0,723	0,339	2,131	0,034	1,05	0,95
	Model fit: adjusted *R*^2^ = 0.0903, *F*_(6, 392)_ = 7.58, *p* value = < 0.001						
Marginal	(Intercept)	8,242	0,114	72,124	< 0.001		
	Education (University)	0,541	0,191	2,834	0,005		
	Model fit: adjusted *R*^2^ = 0.0174, *F*_(1, 397)_ = 8.03, *p* value = 0.0048						
VSABTH-T total score	(Intercept)	114,381	2,295	49,832	< 0.001		
	Age	−0,186	0,046	−4,015	< 0.001	1,03	0,97
	Occupation (Officer)	4,375	1,890	2,315	0,021	1,02	0,98
	Education (University)	3,456	1,612	2,145	0,033	1,02	0,98
	Model fit: adjusted *R*^2^ = 0.0672, *F*_(4, 394)_ = 8.17, *p* value = < 0.001						

VSABTH-T, Valencia scale of attitudes and beliefs toward; *p*-value < 0.05; Statistical Significant β, coefficient estimate; SE, standard error; *t, t* statistic tolerance < 0.20 or VIF > 5: indicates high multicollinearity.

Regression analyses predicting SSS scores indicated that age predicted physiological suggestibility and peer conformity (*B* = 0.018, *p* = 0.003). Prior hypnosis experience predicted peer conformity (*B* = 0.906, *p* = 0.041). Retirees had lower conformity (*B* = −0.765, *p* = 0.002) and persuasibility (*B* = −0.888, *p* = 0.001). Income level predicted physiological suggestibility (*B* = −1.094, *p* = 0.017), and hypnosis knowledge predicted susceptibility to influence (*B* = 1.833, *p* = 0.019; Table 6).

**Table 6:** Multiple regression models predicting SSS scores.

**Dependent variable**	**Predictors**	**β**	**SE**	***t***	***p*-value**	**Vif**	**Tolarence**
Peer conformity	(Intercept)	6,632	0,255	26,051	< 0.001		
	Hypnosis experience (yes)	0,906	0,443	2,047	0,041	1,01	0,99
	Age	0,018	0,006	3,026	0,003	1,49	0,67
	Occupation (Emekli)	−0,765	0,247	−3,095	0,002	1,51	0,66
	Occupation (Health professional)	0,410	0,205	2,004	0,046	1,10	0,91
	Model fit: adjusted *R*^2^ = 0.0672, *F*_(4, 394)_ = 8.17, *p* value = < 0.001						
Physiological suggestibility	(Intercept)	13,091	0,645	20,296	< 0.001		
	Income level (intermadiate)	−1,094	0,456	−2,398	0,017	1,01	0,99
	Age	0,033	0,013	2,511	0,012	1,01	0,99
	Occupation (tradesman)	2,329	1,057	2,204	0,028	1,00	1,00
	Model fit: adjusted *R*^2^ = 0.0672, *F*_(4, 394)_ = 8.17, *p* value = < 0.001						
Persuadability	(Intercept)	14,431	0,122	118,632	< 0.001		
	Occupation (Retired)	−0,888	0,270	−3,288	0,001		
	Model fit: adjusted *R*^2^ = 0.0672, *F*_(4, 394)_ = 8.17, *p* value = < 0.001						
Influence of others	(Intercept)	8,167	0,068	120,928	< 0.001		
	Hypnosis knowledge (high)	1,833	0,779	2,354	0,019		
	Model fit: adjusted *R*^2^ = 0.0672, *F*_(4, 394)_ = 8.17, *p* value = < 0.001						

SSS, The Short Form of The Suggestibility Scale; *p*-value < 0.05; statistical significant β, coefficient estimate; SE, standard error; *t, t* statistic tolerance < 0.20 or VIF > 5: Indicates high multicollinearity.

## 5 Discussion

The findings of this study indicate that age plays an important role in shaping attitudes and behaviors toward hypnosis, with older participants showing less favorable overall attitudes, including lower levels of fear, perceived control, and cooperation, while demonstrating slightly greater physiological suggestibility. In contrast, hypnotizability as a whole did not vary with age. Gender and educational status showed no meaningful associations with either attitudes toward hypnosis or hypnotizability. Occupational differences were evident: officers exhibited more positive attitudes, particularly in cooperation, fear, and assistance-related domains, whereas unemployed individuals showed stronger concerns about control. These groups also differed in components of hypnotizability, with unemployed participants displaying greater physiological responsiveness and officers showing higher persuadability, although total hypnotizability did not differ across occupations. Income level contributed additional variation, as lower-income individuals were more physiologically suggestible, higher-income individuals were more cooperative, and those in the middle-income range reported the most positive overall attitudes, despite similar levels of hypnotizability across income groups. Prior knowledge or experience with hypnosis did not meaningfully influence attitudes or hypnotizability, apart from a single attitude subdomain. Attitudes toward hypnosis and hypnotizability were not related at the total-score level, though several very weak relationships emerged among specific subcomponents.

Hypnosis can influence a person's awareness, bodily sensations, emotions, thoughts, memories, or behaviors. It is a structured procedure designed to facilitate specific changes in these domains. In other words, hypnosis aims to produce lasting, subconscious changes that are acceptable to the individual (Mouheb et al., 2025). During hypnosis, changes occur in several brain regions, such as the limbic system, which plays a central role in emotional regulation. In addition, regions including the diencephalon, basal ganglia, the insular cortex, and areas of the sensory and motor cortices are affected (Zahedi et al., 2024). For these reasons, hypnosis may be used as a supportive therapeutic method in a wide range of conditions (Yerzhan et al., 2025; Walter et al., 2025).

Although Häuser et al.'s meta-analyses indicate that hypnosis is a safe and non-harmful therapeutic technique, concerns about its use may still arise within the general public, among patients scheduled to undergo the procedure, and even among healthcare professionals (Häuser et al., 2016; Krouwel et al., 2017; Desai et al., 2011; Lynn et al., 2020). Hesitations regarding hypnosis may stem from concerns about its clinical efficacy, the extent to which it can provide therapeutic benefits, individual variations in hypnotic responsiveness and suggestibility, and the processes involved in induction, as well as issues related to maintaining control and memory during the hypnotic state (Zahedi et al., 2024). The most prominent concern is that, because hypnosis is typically performed with the eyes closed, it is often mistakenly assumed that the individual enters a sleep-like state, experiences altered consciousness, or even loses control. Additional common concerns include the belief that an individual may be compelled to perform actions against their will while in a hypnotic state, as well as the fear of being unable to emerge from hypnosis. Other concerns related to hypnosis include the belief that a person may be compelled to recall all past traumatic or distressing experiences during the procedure, as well as the misconception that some individuals are inherently incapable of being hypnotized (Krouwel et al., 2017; Desai et al., 2011; Lynn et al., 2020; Tastan et al., 2019). Some individuals may perceive hypnosis as a supernatural phenomenon and may harbor concerns or apprehensions about its practice (Tastan et al., 2019). The findings of this study indicate that participants share similar concerns regarding hypnosis.

An individual's response to hypnotic procedures is strongly influenced by their attitude toward hypnosis, their belief in its effectiveness, and their inherent level of hypnotic susceptibility (Thompson et al., 2019; Walter et al., 2025; Häuser et al., 2016). Attitude refers to a predisposition that an individual forms toward a situation or event, encompassing emotional, cognitive, and behavioral components. The development of such an attitude requires a certain level of awareness of the situation or event (Thompson et al., 2019). The concept of belief encompasses both true and false propositions. It represents a psychological state in which an individual accepts or rejects a given idea, often through an intuitive or non-analytic evaluative process (Dell, 2021). An individual's capacity to respond to physiological, emotional, cognitive, or behavioral suggestions during hypnosis is referred to as hypnotic susceptibility. Higher levels of susceptibility enable individuals to respond more effectively to hypnotic suggestions, thereby increasing the overall efficacy of the hypnosis process (Geagea et al., 2023a).

A review of the literature shows that numerous studies have been conducted to evaluate attitudes and behaviors toward hypnosis, levels of awareness, and hypnotizability (Palsson et al., 2019; Krouwel et al., 2017; Verhoef and Sutherland, 1995; Jafari et al., 2021; Demir-Dora et al., 2020; Bansal et al., 2020; Molina-Peral et al., 2020; Capafons et al., 2008; Coldrey and Cyna, 2004; Koep et al., 2020; Green et al., 2006; Madan, 2015; Martín et al., 2010). A cross-sectional study conducted in the United States with 1,000 participants found that 38.6% held a positive opinion of clinical hypnosis, 48.4% were neutral, and 12.8% expressed a negative opinion. Among participants, 7.6% had previously undergone hypnosis treatment, with 63.1% reporting some benefit. Of those who had not received hypnosis, 54.9% indicated willingness to consider such treatment. Overall, 45.6% of participants believed there was moderate to strong scientific evidence supporting hypnosis. Notably, attitudes toward hypnosis were consistent across gender, age, and education levels. Additionally, the majority preferred that hypnosis be performed by health professionals. A key strength of the study was that participants were unaware the survey pertained to hypnosis prior to responding, reducing potential bias (Palsson et al., 2019). In a comprehensive review conducted by Krouwel et al., which examined English-language articles published between 1996 and 2016 on public perceptions of hypnosis, it was found that hypnotherapy was generally regarded as effective for addressing psychological problems. The reviewed studies also indicated that participants expressed willingness to undergo hypnosis when it was administered by qualified practitioners (Krouwel et al., 2017). In this study, most participants reported negative concerns regarding hypnosis and indicated that they did not wish to undergo a hypnotic experience.

In a study conducted in Canada to assess general practitioners‘ opinions and practices regarding complementary and alternative medicine, acupuncture, chiropractic, and hypnosis were identified as the most useful methods. Among the participants, 56% reported perceiving these methods as beneficial, 54% indicated that they referred patients for such treatments, and 16% stated that they personally applied one of these methods in their practice (Verhoef and Sutherland, 1995). Conversely, another cross-sectional study conducted among healthcare professionals revealed that, although 79% held a positive attitude toward complementary medicine, 73.6% demonstrated limited knowledge, and hypnosis was among the least preferred methods, with only 4.3% reporting its use. A significant relationship was observed between participants' level of education and both their likelihood of recommending and their actual use of these methods (Jafari et al., 2021). A cross-sectional study conducted among medical students in Türkiye found that only 9–16% had knowledge of complementary medicine practices. Among these, acupuncture was the most recognized, while prolotherapy was the least known; however, hypnosis emerged as the practice that generated the greatest curiosity and interest (Demir-Dora et al., 2020). This study is the first to be conducted with physical therapy patients and therefore differs from previous research in the field. However, most of the participants were healthcare professionals, officers, or retirees. Despite this demographic profile, interest in hypnosis, positive attitudes toward the practice, and willingness to undergo hypnosis were lower compared with findings from earlier studies. However, the regression analysis showed that health professional and officer participants tended to have more positive attitudes.

A cross-sectional study conducted among adults in India using a 26-item questionnaire showed that 82.5% of participants held a positive attitude toward clinical hypnosis, despite demonstrating low levels of awareness about the practice. A positive correlation was identified between awareness scores and attitude scores. Additionally, four out of the five participants who had previously undergone clinical hypnosis reported finding it beneficial and expressed a desire to experience it again (Bansal et al., 2020). In another study conducted by Molina-Peral et al. involving Portuguese students, individuals without prior exposure to hypnosis exhibited higher negative attitude scores, whereas those with previous experience demonstrated higher positive attitude scores. The study further reported that positive attitudes toward hypnosis were particularly elevated when the procedure was administered by a psychologist (Molina-Peral et al., 2020). Copafons et al.'s study involving student samples from Spain, the United States, Portugal, and Romania examined how personal experience with hypnosis and knowledge sources influenced beliefs and attitudes toward hypnosis. The findings indicated that participants who had previously undergone hypnosis and/or based their understanding on scientific sources generally demonstrated more positive attitudes toward hypnosis and obtained higher scores on measures reflecting accurate beliefs about the practice (Capafons et al., 2008). Coldrey et al.'s study examining anesthesiologists' knowledge of and attitudes toward hypnosis found that 63% of participants had below-average knowledge, while approximately half expressed support for its practice. Furthermore, participants with prior experience in hypnosis demonstrated more positive attitudes toward it (Coldrey and Cyna, 2004). In the study conducted by Koep et al., a significant correlation was identified between attitudes and behaviors toward hypnosis and scores on the Elkins Hypnotizability Scale among 91 undergraduate students. In contrast, the present study did not demonstrate a significant relationship between the VSABTH-T and the SSS (Koep et al., 2020). In this study, contrary to the findings reported in the literature, no significant correlation was observed between attitudes and behaviors toward hypnosis and hypnotizability scores. The correlations identified between the subparameters were also exceedingly weak. Although the regression analysis showed that hypnosis experience and knowledge had an effect, the size of this effect was quite small. This discrepancy may be explained by the fact that most participants had never previously experienced hypnosis and possessed very limited knowledge about the practice.

The study performed by Green et al. involving participants from multiple countries has indicated that, despite minor variations in responses, attitudes, and beliefs about hypnosis are largely consistent across different cultures. Additionally, a positive relationship has been observed between individuals' susceptibility to hypnosis and their positive attitudes and beliefs toward it. It has been suggested that fostering more positive attitudes may enhance responsiveness to hypnotic suggestions (Green et al., 2006). In this study, no significant association was identified between the VSABTH-T and the SSS. Additionally, these scores showed no associations with gender, education level, prior hypnosis experience, or level of hypnosis knowledge. In contrast, significant associations were observed with age, occupation, and income level.

A study investigating attitudes and beliefs about hypnosis, as well as its current use by psychologists, counselors, and physiotherapists in Australia, found that 58.7% of participants had prior experience with hypnosis, while only 30.6% actively practiced it. Overall, participants demonstrated generally positive attitudes and accurate beliefs regarding hypnosis. However, the authors emphasized that the findings may not be generalizable, as a majority of individuals who received online invitations to participate did not respond (Madan, 2015). A study conducted in Cuba on health professionals who had either attended or not attended hypnosis classes found that both groups initially held similar misconceptions about hypnosis and memory. However, participants who attended the hypnosis classes showed a reduction in these misconceptions and demonstrated more positive attitudes toward hypnosis compared to those who did not participate in the classes (Martín et al., 2010). This study revealed that participants' knowledge about hypnosis was considerably limited. If future initiatives aim to enhance public understanding and firsthand experience of hypnosis, positive shifts in attitudes and behaviors toward hypnosis may occur, consistent with findings reported in previous research.

## 6 Strengths and limitations

The major strength of this study is that it is the first study conducted in a physical therapy patient population. The major limitation of the study is that, because concepts such as attitude, behavior, and hypnotizability are abstract, assessments were based on questionnaires and participant self-reported information, and there is no objective measurement tool. Additionally, most participants had neither prior knowledge of nor experience with hypnosis. A sample with greater familiarity and experience with hypnosis might have yielded more robust and meaningful correlations. Because this study was conducted exclusively on a physical therapy patient population, the findings cannot be generalized to broader populations. Additionally, the very low correlations observed between the evaluation scores and their sub-parameters, as well as the small explanatory power detected in the regression analyses, further limit the generalizability of the results. More comprehensive studies are needed, particularly those that consider the wide range of psychological and cultural factors that may influence attitudes toward hypnosis and levels of hypnotic suggestibility. In addition to this, the study methodologically did not assess the pre- or post-hypnosis information or the pre- and post-hypnosis experience. Had such a methodology been adopted, a correlation between the VSABTH-T and the SSS could have been detected. Therefore, future multicenter studies with pre- and post-hypnosis assessments should be designed with diverse populations. Another limitation of the study was the absence of a published protocol prior to reporting the outcomes, which is important for promoting research transparency and minimizing publication bias.

## 7 Conclusion

Participants showed limited knowledge of hypnosis and largely negative attitudes, marked by fear, loss-of-control concerns, and reluctance. Older individuals held more negative views despite greater physiological suggestibility, while most other demographic factors showed little influence. Although overall attitudes were not linked to hypnotizability, small but significant associations appeared in specific subdimensions. These findings highlight the complex relationship between beliefs and hypnotic responsiveness and emphasize the need for better education and larger, well-designed studies to address misconceptions and evaluate outcomes more effectively.

## Data Availability

The original contributions presented in the study are included in the article/supplementary material, further inquiries can be directed to the corresponding author.
